# The costs and benefits of water fluoridation in NZ

**DOI:** 10.1186/s12903-017-0433-y

**Published:** 2017-11-28

**Authors:** David Moore, Matthew Poynton, Jonathan M. Broadbent, W. Murray Thomson

**Affiliations:** 1 Sapere Research Group Limited, PO Box 587, Wellington, New Zealand; 2 Sir John Walsh Research Institute, School of Dentistry, The University of Otago, PO Box 56, Dunedin, New Zealand

**Keywords:** Water fluoridation, Cost-effectiveness analysis, Health economics, Oral health, Public health

## Abstract

**Background:**

Implementing community water fluoridation involves costs, but these need to be considered against the likely benefits. We aimed to assess the cost-benefit and cost-effectiveness of water fluoridation in New Zealand (NZ) in terms of expenditure and quality-adjusted life years.

**Methods:**

Based on published studies, we determined the risk reduction effects of fluoridation, we quantified its health benefits using standardised dental indexes, and we calculated financial savings from averted treatment. We analysed NZ water supplies to estimate the financial costs of fluoridation. We devised a method to represent dental caries experience in quality-adjusted life years.

**Results:**

Over 20 years, the net discounted saving from adding fluoride to reticulated water supplies supplying populations over 500 would be NZ$1401 million, a nine times pay-off. Between 8800 and 13,700 quality-adjusted life years would be gained. While fluoridating reticulated water supplies for large communities is cost-effective, it is unlikely to be so with populations smaller than 500.

**Conclusions:**

Community water fluoridation remains highly cost-effective for all but very small communities. The health benefits—while (on average) small per person—add up to a substantial reduction in the national disease burden across all ethnic and socioeconomic groups.

**Electronic supplementary material:**

The online version of this article (10.1186/s12903-017-0433-y) contains supplementary material, which is available to authorized users.

## Background

Millions of school and work hours are lost to oral disease globally [1, 2], and it is the fourth most expensive disease category [3]. Increasing numbers of NZ adults are now keeping their teeth [4], which means that their risk of dental caries is now greater than ever before. It is the most common chronic disease [3] and markedly affects sufferers’ day-to-day lives. It continues through life, with about one newly affected tooth surface per year in the average person [5]. Anyone with teeth (whether child or adult) is at risk of the disease, although dental caries rates in children have declined in developed countries over the last four decades [6]. The decline is generally attributed to the introduction of fluoride in toothpaste, water and salt [7], although the proportion due specifically to water is unclear [8].

High by international standards [4], caries rates in NZ are disproportionately higher in disadvantaged subgroups, with Māori, Pacific peoples, and those in deprived areas having more untreated caries and missing teeth, and greater impacts on their quality of life [9]. Māori adults have 10% more teeth affected by caries; Māori children have 50% more primary teeth and 80% more permanent teeth affected, and they are 30% less likely to be caries-free. Māori also report poorer access to dental services [4]. In 2013, 43% of Māori but 57% of non-Māori children in Year 8 were reported to be caries-free [10].

Fluoride occurs naturally in most water sources, although NZ’s natural levels (<0.2 ppm) are low by international norms. The Ministry of Health recommends adjustment of the fluoride concentration to between 0.7 and 1.0 ppm [11]. Since the late 1940s, many countries with suboptimal natural levels have introduced fluoride to their water supplies, a process known as community water fluoridation (CWF). This is now done in about 25 countries [12], with much higher coverage rates in developed nations (such as 74% in the USA [13]). Fluoridation began in NZ in 1954 and expanded rapidly through the 1960s. Some 3.8 million New Zealanders (85%) are supplied with reticulated water, and 56% of those receive fluoridated water [14]. Currently, 39^1^ of 66 territorial local authorities do not adjust the fluoride level in their water supplies. As a means of promoting the practice, the Ministry of Health may subsidise their capital costs, considering each case as it arises.

There have long been claims and counter-claims about the side-effects of CWF, but there are none to speak of at NZ levels of concentration [14]. Recent concerns about side-effects have centred on cancer (most notably osteosarcoma) and children’s cognitive development. These and other concerns have been extensively reviewed in a number of jurisdictions in recent years, and they have found to be neither realistic nor significant [14].

Numerous NZ and international studies have shown that CWF reduces caries, but they have reported different levels of benefit. This may be explained in part by the reduction in caries over time, improvements in other forms of prevention, differences in study populations, and differences in measurement—and adjustment for duration—of exposure to water fluoridation.

Implementing CWF involves costs, but these need to be considered against the likely benefits, a process known as cost-effectiveness analysis. For example, expenditure on capital and running costs for fluoridating a city of 100,000 might be more than offset by the averted costs of dental caries and its sequelae, but that may not be the case for smaller communities. An earlier CEA study of CWF in NZ [15] found CWF to be cost-saving for communities >1000, but the analysis was limited to child data from a single study, with no benefits assumed for anyone older than 35, and the equipment and plant cost assumptions were insufficiently detailed. A more recent NZ analysis [16] supported the findings of the earlier analysis [15] but, because it under-estimated the averted dental restoration costs, the extent of the cost-effectiveness remains unclear. Sound public health practice requires periodic re-evaluation of interventions’ benefits and costs. Accordingly, the aim of this study was to provide an updated evaluation of the benefits and costs of water fluoridation in NZ.

## Methods

### Determining the effectiveness of CWF

We obtained estimates of the caries-risk-reduction benefits of CWF from the literature. Most studies have used children. The NZOHS [4] provided the most valid estimates for NZ children. It included 987 children, used a stratified random sample, and had high-quality data recording and consistency in reporting. Children living in fluoridated areas had 40% lower caries experience than others (adjusted for age, sex, ethnicity and deprivation). Other NZ studies generally support this. In a study of Auckland 9-year-olds [17], those who had lived all of their lives with CWF were under half as likely as those who had never done so to have experienced caries, while those who had spent part of their lives with CWF also had lower odds. A study of 436 Southland 9-year-olds found the rate of caries in fluoridated areas to be half that in unfluoridated areas [18]. A study of 1996 routinely collected data on 5- and 12-year-olds from fluoridated Wellington and nonfluoridated Christchurch found the former to have substantially lower caries experience [19]. These findings are mirrored overseas. The York report (on 26 studies from 1951 to 1999) is the key meta-analysis [20]. It focused on children and found a 38% reduction in caries in areas with water fluoridation. All included studies were of evidence level B (moderate quality and moderate risk of bias) or higher. Fluoride levels in the fluoridated areas ranged from 0.7 to 1.2 ppm, with most unfluoridated areas under 0.2 ppm (as in NZ). A more recent meta-analysis of studies from 1990 to 2010 reported reduction estimates of 30–59%, in line with the York report [21]. For the current study, we used the NZOHS estimate of 40% reduction for children.

The first systematic meta-analysis to report water fluoridation benefits for adults provided two sets of findings, one of which was restricted to five studies (all cross-sectional) that had no heterogeneity issues [22]. We consider only this set, which found a reduction in caries of 27%, consistent with the Australian National Survey of Adult Oral Health (NSAOH): reductions of 21% for ages 18–44 and 30% for ages 45+ (determined at surface level); 10% for ages 18–44 and 11% for ages 45+ (determined at tooth level) [23]. The surface-level findings are more likely to accurately represent the effects of fluoridation. The methods used were the same as the NZOHS, but information was also collected about residential history and participants were categorised by their length of time spent living in areas with CWF, with estimates adjusted for that, along with age, region, country of birth, reason for dental visits, tooth-brushing frequency, annual income and education level. Given the methodological soundness of the NSAOH, its rigorous adjustment for confounders, the similarity of fluoride levels in Australian and NZ water supplies, and similar levels of caries in the two countries, we considered it the most robust estimate applicable to NZ adults.

### Forecasting the health benefits of fluoridation

Our time horizon was 20 years (the expected life of capital investment in water plants), with relative risk reduction (RRR) estimates of 40% for under-18 s, 21% for ages 18–44 and 30% for ages 45+. We first assumed the reported caries experience for a given age group to be halfway between that with and without fluoridation, because approximately half of New Zealanders live in areas with fluoridation. This will likely result in a small overestimation of the time exposed to fluoridated water supplies and so underestimate caries experience, since many New Zealanders currently with fluoridated water have spent parts of their lives without it. We applied the RRRs to the NZOHS dental caries rates (Additional file 1: Table S1). Second, we disaggregated the caries experience estimate to untreated carious and missing or filled surfaces to apply the cost of treatment (and estimate averted treatment costs). We assumed that CWF has the same relative effect on each of the three outcomes of caries (Additional file 1: Table S2). Third, we estimated the approximate annual rate of caries experience with and without fluoridation by using the age-group-specific estimates from the NZOHS, with the assumption that no period or cohort effects were operative. For example, we estimated the average number of filled tooth surfaces for a 45–54-year-old with exposure to water fluoridation to be 25.5, which is 13.1 more than for a 35–44-year-old. Thus, the annual rate of filled surfaces for those aged 45–54 with exposure to water fluoridation is 1.3 (13.1 filled surfaces over 10 years; Additional file 1: Table S3). In cases where there is a drop in a measure of caries as the age group increases, we assume an annual rate of zero increase (in some cases, teeth that have previously had untreated caries or a filling will become missing). In order to simplify the calculations, we have assumed that all of the caries experience seen in the permanent teeth of 12–17-year-olds occurred during that age band. This simplification will likely slightly underestimate caries in the 5–11 group and slightly overestimate it in the 12–17 group. We estimated an average of 11 years age difference between the 75+ age group and the 65–74 group, assuming the average 75-year-old to live until 81 (based on Statistics NZ life tables). Fourth, we applied Statistics NZ population forecasts to the above annual probabilities, to estimate the total number of affected tooth surfaces with and without CWF for the period 2016 to 2035. We also estimated the total number of affected teeth, by applying the ratio of surfaces affected per tooth, as represented in the NZOHS dmfs/DMFS and dmft/DMFT scores. These rates were applied to the dentate population only (edentulism prevalence estimates for each age group were obtained from the NZOHS; Additional file 1: Tables S4 and S5).

We used the Statistics NZ 2011 base 50th percentile population projection. We applied the above rate estimates to the population in residences served by a public water supply (currently 3.8 million people). The NZ population is estimated to reach 5.3 million by 2035; accordingly, we estimated that 4.5 million people will have a public water supply, at an average of 4.2 million over the 20 years.

### Averted cost assumptions

We discounted future benefits back to today’s dollars. A discount rate of 3.5% p.a. was applied to benefits and costs; this is the standard discount rate applied by PHARMAC in economic analysis [24], and its use facilitates comparison of findings with those for other health interventions. The discount rate is a real rate of return.

Our costing of treatment for adults was based on the following averages from the NZ Dental Association’s 2013 fee survey: $200 for a single extraction with local anaesthetic; $219 for a composite restoration (Class II); $282 for a multi-surface composite; $143 for a one-surface amalgam; and $189 for a two-surface amalgam [25]. We applied the cost of a single extraction for each missing tooth. For the cost of an averted restoration, we used a weighted average of the multi-surface composite and two-surface amalgam costs (at $247 per filled tooth). We estimated a saving of $90 where water fluoridation is estimated to reduce the number of filled surfaces but not avoid a restoration. This estimate is based on the difference in costs of single and multi-surface restorations, and is based on the same ratio of composite and amalgam as above. We assumed different replacement rates for different types of dental restoration, given that the frequency with which a restoration has to be replaced depends on the material used. Silver amalgams are more durable (typically lasting 13 years), while composites (tooth-coloured) are more aesthetic but are less durable (typically lasting 5 years) [26]. At the General Dental Practice clinic in the University of Otago Faculty of Dentistry during 2009, 62% of restorations placed were composite, with the remaining 38% being amalgam. We used these rates in our analysis, with an estimated average cost of an adult restoration of $247 and an average time for replacement of 7.9 years.

Basic oral health care in NZ is free for children under 18. Dentists are remunerated by the Government at the following rates: $58.03 for a single extraction with local anaesthetic; $74.36 for a simple non-metallic restoration in an anterior tooth; $99.99 for a multi-surface non-metallic restoration; $62.56 for a one-surface restoration in a posterior tooth; and $82.05 for a two-surface restoration in a posterior tooth. We applied the cost of a single extraction for each missing tooth. For the cost of an avoided filling, we used a weighted average of the four listed types of restoration (calculated to be $84). We used the same ratio of composite to amalgam as for adults, and we assumed that 40% of restorations placed were single-surface fillings and 60% were multi-surface fillings (based on NZ children having an average of 1.6 surfaces affected by each filling).

Our costings were conservative, since they included only basic treatment, whereas more expensive treatment items (such as dental crowns, dental bridges, endodontic treatment and implants) are also likely to be averted to some degree by fluoridation.

Two studies were found to have estimated the impact of water fluoridation on caries-associated hospitalisations of young children: a Dunedin study reported a 73% lower rate for children under 6 [27], while an English study reported 48% for children aged 1 to 4 [28]. Our analysis used the latter, more conservative finding. We estimated that, in the year ending June 2012, there had been 2918 NZ admissions for children aged 0 to 4 requiring treatment for dental caries. This was based on hospital admission data recorded in the National Minimum Dataset [29] where the primary diagnosis relates to dental caries. We identified caries using the ICD 10 codes K02 (“dental caries”) and K04 (“diseases of pulp and periapical tissues”). The estimated cost of those admissions was $5.6 million.

### Calculating quality-adjusted life years

We assessed the health benefits of water fluoridation by estimating the quality-adjusted life years (QALYs) gained. A particular benefit of QALY analysis is that it allows easier comparison with other health initiatives, such as pharmaceutical investments or screening.

Recent years have seen a considerable number of studies of the effect of dental caries on quality of life (QoL). Many have used the short-form Oral Health Impact Profile (OHIP-14) [30], a measure of the impact of poor oral health. However, it does not capture the trade-off between improved QoL and improved life expectancy and so cannot be used to derive utility values in QALY estimates. Formulae have been derived for converting OHIP-14 data to QoL values [31], but the absence of estimates of CWF’s impact on measures such as the OHIP-14 precluded our using them. Our approach was similar to the standard method of estimating QALYs in cost-utility analyses: we assigned differing QoL scores (assuming the QALY loss is similar to DALY loss reported in the NZ Burden of Disease Study [32]) to those with low caries (0–2 DMFT, QoL = 1.000), moderate caries (3–11 DMFT, QoL = 0.999) and high caries (12+ DMFT, QoL = 0.997); we also included edentulism (no teeth, QoL = 0.997; see Additional file 1: Table S6.) A QoL value of 1.000 represents full quality of life and a QoL value of 0.000 is equivalent to being dead. The inference of using a QoL value of 0.997 for the high caries group is that, on average, people would be willing to give up one day each year in order to be restored to full QoL. We estimated the proportion of people in each of these groups based on their water fluoridation status and age group (from NZOHS data adjusted for age, sex, ethnicity and deprivation). In order to estimate the QALY gain from water fluoridation, we multiplied the difference in the proportion of people in health states, forecast the age distribution, and then applied the QoL values.

### Measuring the costs of fluoridation

Costs include: (a) set-up and capital; (b) the ongoing supply of fluoride; and (c) ongoing operational costs. NZ legislation defines five sizes of water supply: neighbourhood (serving up to 100 people); small (101–500); minor (501–5000); medium (5001–10,000); and large (over 10,000). We examined the three cost categories for each of these plant sizes.

- Set-up and capital costs.

Three combinations of fluoride chemical and feed system are currently in use (or could possibly be used) in NZ. The choice impacts all three aspects of the financial costs. We used two primary data-sets. The first, covering engineering-based costs, was an estimation of water fluoridation costs by water engineering company CH2M Beca [33]. We supplemented this with cost information from nine district councils (because Beca made no cost estimate for ‘large’ plants); these data provided cost estimates per plant. The second, comprising water supply data, was provided by the Institute of Environmental and Scientific Research; these data provided estimates of the number of plants of each size and the amount of fluoride required. There is a complex relationship between the supply and usage of water. A single water plant can supply multiple communities (even multiple local authorities), and a single community can be served by a number of water plants. Furthermore, the data are incomplete. Attribution of costs to district councils can therefore be difficult. Accordingly, we made a number of important assumptions. First, Beca provided the capital cost of fluoridation equipment as a range reflecting different existing configurations. We used the midpoint of each range. We assumed the lowest cost combination of capital and fluoride type for each plant (although some councils do not take this approach).

(b) The ongoing supply of fluoride.

The ongoing cost of fluoride compound was calculated from the average amount of water supplied by each plant and the price of fluoride. The type of fluoride used was determined by the water treatment plant size.

(c) Ongoing operational costs.

We assumed that operating costs are the same for medium and large plants; that is, ongoing costs differ only according to the volume of water. Our cost estimates assumed fluoridation at each plant, although, in some cases, water from multiple plants is piped to a single point and then fluoridated. Water supply data are recorded in cubic metres per day. Many plants (generally larger ones) record this information. Where they did not, we estimated the amount of water by applying the average water usage per person to the population supplied by the plant. We calculated the cost of fluoride per unit of water used (m^3^/day), based on Beca data [33]. The annual cost of fluoride per m^3^/day was calculated as the annual cost divided by the average water flow (m^3^/day). A summary of our analysis is presented in Table 1.

**Table 1:** Estimated cost of water fluoridation by plant size

Plant size	Population	Fluoride chemical	Total capital set-up costs	Annual operating costs	Annual fluoride supply cost per m^3^/day
Neighbourhood	<100	Sodium fluoride (NaF)	$112,500	$6700	$3.57
Small	101–500		$117,500	$7100	$3.46
Minor	501–5000		$170,000	$8200	$3.41
Medium	5001–10,000	Fluorosilicic acid (FSA)	$202,500	$8900	$1.25
Large	10,001+		$347,004	$8900	$1.25

We varied the discount rate between 0 and 8% p.a., based on NZ Treasury guidelines [33]. We varied the efficacy of water fluoridation by −23% and +23% for all age groups, based on one standard deviation of the estimated impact of water fluoridation in adults (calculated by us from values reported for the DFS measure [23]).

## Results

### Cost-effectiveness

For neighbourhood and small plants, the cost of fluoridation is greater than the estimated cost offsets from averted dental costs (Table 2). For ‘minor’ through to ‘large’ plants, there is a net cost saving. For a ‘large’ plant supplying 50,000 people, the cost offsets are over 20 times the cost of fluoridation. The break-even point appears to be reached by ‘minor’ plants supplying a population over 500.

**Table 2:** Costs and savings of fluoridation by plant size (20-year horizon)

Plant size	Population used for estimate	Fluoridation cost	Dental care cost	Net cost^a^
Neighbourhood	50	$212,000	$19,000	$193,000
Small	250	$228,000	$94,000	$134,000
Minor	2500	$348,000	$939,000	-$591,000
Medium	7500	$397,000	$2,818,000	-$2,421,000
Large	50,000	$900,000	$18,785,000	-$17,885,000

^a^A negative value indicates a net saving

We estimated the following averted treatment if CWF were to be implemented at all plants supplying populations over 500: 459,000 teeth with untreated caries; 4,068,000 extractions; and 3,361,000 restorations. We estimated the national net saving from universal fluoridation of supplies for populations over 500 over 20 years to be $1401 million (cost of fluoridation $177 million, cost offset $1578 million). The cost would be incurred by local government (with a small proportion subsidised centrally). The saving to the health budget would be $149 million. Most of the savings would come from a $1428 million reduction in private dental care expenditure. The average cost of CWF was estimated to be over four times higher in areas that do not currently have fluoridated water (Table 3). This is because those areas tend to have smaller plants. The cost offsets were estimated to be the same for areas currently with and without fluoridated water. Our sensitivity analysis (Table 4) showed that the model was most sensitive to assumptions on the discount rate, the efficacy of water fluoridation and the costs of treating caries. In all of our scenarios, CWF is cost-saving.

**Table 3:** Costs by current fluoridation status (20-year horizon)

Comparator	Intervention	Fluoridation cost	Dental care cost	Net cost^a^
No fluoride	Current fluoride	$32 m	-$790 m	-$758 m
Current fluoride	All fluoride	$144 m	-$788 m	-$644 m
No fluoride	All fluoride	$177 m	-$1578 m	-$1401 m

^a^A negative value indicates a net saving

**Table 4:** One-way sensitivity analysis (20-year horizon)

Variable	Base case	Updated	Net cost
Base Case			-$1401 m
Efficacy of fluoridation			
Average risk reduction caries	24%	19%	-$1002 m
		30%	-$1836 m
Cost of fluoridation			
Plant capital costs	100%	50%	-$1444 m
		200%	-$1317 m
Plant maintenance costs	100%	50%	-$1427 m
		200%	-$1350 m
Cost of fluoride	100%	50%	-$1422 m
		200%	-$1361 m
Cost offsets			
Cost of a filling^a^	$220	$176	-$1213 m
		$264	-$1589 m
Proportion of fillings that are composite	62%	40%	-$1196 m
		70%	-$1458 m
Cost of an extraction	$177	$141	-$1284 m
		$212	-$1519 m
Discount rate			
Discount rate	3.5%	0.0%	-$2035 m
		8.0%	-$927 m

^a^Average (weighted) cost for adults and children

### Quality-of-life benefits

The common approach to reporting QALYs is to report an incremental cost-effectiveness ratio, which is the net additional cost divided by the net additional QALYs. We have chosen to report the QALYs separately from the costs, since water fluoridation is cost-saving for minor, medium and large plants, meaning that there is no incremental cost per QALY. We estimated that the provision of fluoridation over 20 years to all reticulated water supplies supplying populations of 500 or more would result in 8800 to 13,700 QALYs gained. The average health benefit per person would be between 0.002 and 0.003 QALYs (discounted; that is, approximately equivalent to an additional 1 to 1.5 days of life at full quality). For each million dollars invested, we estimated 50 to 78 QALYs gained and $9 million in savings.

## Discussion

This study investigated the cost-effectiveness of CWF in NZ and found the procedure to be highly cost-effective, with the benefits in terms of averted suffering and dental care costs discernible for communities as small as 500 people. In short, there is a nine times pay-off, with savings over two decades: the costs fall on territorial local authorities but the benefits are largely enjoyed by private individuals (and the health system in a small way).

Any cost-effectiveness analysis relies on a number of assumptions. We are confident of the robustness of ours, given that they were based on peer-reviewed, published epidemiological data and actual cost data supplied by local authorities and the NZ Dental Association. The values we assign to the efficacy of fluoridation are in line with the best available evidence, bearing in mind especially the importance of allowing for duration of exposure to fluoridation. Our estimates of averted treatment are cautious, covering only basic care. Our base case, therefore, is a robust conservative assessment of the benefits. QALY gains—though small per person—add up to a substantial reduction of the national disease burden. The CWF finding is in stark contrast to pharmaceuticals, where there is a net cost in funding new medicines [34]and an average return per million dollars of at least 27 QALYs.

Although we expect the relative impact of water fluoridation to be the same across ethnic groups and socioeconomic class, the greater caries experience in Māori, Pasifika and those who are most deprived means that we expect them to have a greater absolute benefit from water fluoridation. For example, Māori children have 80% more permanent teeth affected by caries. Accordingly, the absolute gain for Māori children should be 80% greater. This gain translates to an estimated gain of 0.9 fewer permanent teeth affected by caries, while it would be 0.5 in non-Māori. Several sources of evidence support this position. Community Oral Health Service records show that the reduction in caries for those attending a school with water fluoridation is greater for Māori than non-Māori, in both absolute and relative terms [10]. A 2004 study of Wellington and Christchurch children reported that Māori and those who are most deprived had greater absolute reductions from water fluoridation, while the relative effect was similar for all groups [19]. A 2014 English study found that children living in the most deprived areas experienced the greatest benefits from fluoridation [28]. It is noteworthy though that, in 2000, the York report found mixed evidence and recommended caution due to the inadequate number and quality of studies at that time [20].

In order to compare our cost estimates with others, we estimated the annual net cost savings per person (undiscounted) to be $24. There was a large range in the published analyses we reviewed, from a net saving of $22 down to a net cost of $26 (the latter is an outlier, based on a population size of under 1000) [15, 16, 35–38]. This is unsurprising, given that those analyses assumed a low benefit from fluoridation, tended to exclude the benefits to adults (which actually comprised 90% of our savings) and tended to limit savings to initial restorations.

Water fluoridation in NZ is highly cost-saving nationally. For a health intervention to be cost-saving at all, let alone by a factor of nine, is exceptional; the small proportion of total savings accruing to the health budget alone would cover much of the total cost. There are strong economic grounds for further Government subsidy of CWF to extend the practice beyond the predominantly large urban centres where it currently takes place. CWF is also exceptional in being an untargeted intervention that narrows the usually intractable health gap between different ethnic and socio-economic groups. As such, fluoridation is a significant driver of health equality, an effect that would increase with its expansion.

## Conclusions

Community water fluoridation remains highly cost-effective. The health benefits—while (on average) small per person—add up to a substantial reduction in the national disease burden across all ethnic and socioeconomic groups.

## Additional file

Additional file 1: Six additional tables of data. (DOCX 23 kb)

## Abbreviations

CWF: Community water fluoridation
NZ: New Zealand
QALYs: Quality-adjusted life years

## Footnotes

1 We include three district councils fluoridating less than 10% of their supply.
