# Supplementary material for: The costs and benefits of water fluoridation in NZ
Source: BMC Oral Health. 2017 Nov 28;17:134. doi: 10.1186/s12903-017-0433-y (PMC5704512; doi:10.1186/s12903-017-0433-y)
Supplement: Additional file 1: — Six additional tables of data. (DOCX 23 kb) [file 12903_2017_433_MOESM1_ESM.docx]

Supplementary tables

**Table A. Estimated dental decay experience by age group**

| **Age group** | **With and without fluoridation NZOHS** | **Without fluoridation (our estimate)** | **With fluoridation (our estimate)** |
| --- | --- | --- | --- |
| 2–4 | 1.8 | 2.3 | 1.4 |
| 5–11 | 3.5 | 4.4 | 2.6 |
| 12–17 | 2.7 | 3.4 | 2.0 |
| 18–24 | 6.3 | 7.0 | 5.6 |
| 25–34 | 13.0 | 14.5 | 11.5 |
| 35–44 | 21.0 | 23.5 | 18.5 |
| 45–54 | 50.0 | 58.8 | 41.2 |
| 55–64 | 65.0 | 76.5 | 53.5 |
| 65–74 | 74.0 | 87.1 | 60.9 |
| 75+ | 75.0 | 88.2 | 61.8 |

Age 2–11 measured by dmfs; age 12+ measured by DMFS.

**Table B. Estimated dental decay experience at surface level by age group**

| **Age group** | **Untreated decay** | | **Missing** | | **Filled** | |
| --- | --- | --- | --- | --- | --- | --- |
|  | **W/O** | **With** | **W/O** | **With** | **W/O** | **With** |
| 2–4 | 0.6 | 0.4 | 0.1 | 0.1 | 1.5 | 0.9 |
| 5–11 | 0.6 | 0.4 | 0.4 | 0.2 | 3.4 | 2.0 |
| 12–17 | 0.4 | 0.2 | 0 | 0 | 3.1 | 1.9 |
| 18–24 | 1.0 | 0.8 | 1.0 | 0.8 | 5.0 | 4.0 |
| 25–34 | 2.2 | 1.8 | 3.1 | 2.5 | 10.2 | 8.0 |
| 35–44 | 1.5 | 1.1 | 5.6 | 4.4 | 15.6 | 12.4 |
| 45–54 | 1.5 | 1.1 | 21.2 | 14.8 | 36.5 | 25.5 |
| 55–64 | 1.3 | 0.9 | 27.1 | 18.9 | 48.2 | 33.8 |
| 65–74 | 0.9 | 0.7 | 42.4 | 29.6 | 43.5 | 30.5 |
| 75+ | 0.9 | 0.7 | 48.2 | 33.8 | 38.8 | 27.2 |

Age 2–11 measured by dmfs; age 12+ measured by DMFS.

**Table C. Estimated annual rate of dental decay experience at surface level**

| **Age group** | **Untreated decay** | | **Missing** | | **Filled** | |
| --- | --- | --- | --- | --- | --- | --- |
|  | **W/O** | **With** | **W/O** | **With** | **W/O** | **With** |
| 2–4 | 0.2 | 0.1 | 0.0 | 0.0 | 0.5 | 0.3 |
| 5–11 | 0.1 | 0.1 | 0.1 | 0.0 | 0.7 | 0.4 |
| 12–17 | 0.1 | 0.0 | 0.0 | 0.0 | 0.4 | 0.3 |
| 18–24 | 0.1 | 0.1 | 0.2 | 0.1 | 0.3 | 0.3 |
| 25–34 | 0.1 | 0.1 | 0.2 | 0.2 | 0.6 | 0.5 |
| 35–44 | 0.0 | 0.0 | 0.2 | 0.2 | 0.5 | 0.4 |
| 45–54 | 0.0 | 0.0 | 1.6 | 1.0 | 2.1 | 1.3 |
| 55–64 | 0.0 | 0.0 | 0.6 | 0.4 | 1.2 | 0.8 |
| 65–74 | 0.0 | 0.0 | 1.5 | 1.1 | 0.0 | 0.0 |
| 75+ | 0.0 | 0.0 | 0.5 | 0.4 | 0.0 | 0.0 |

**Table D. Estimated number of tooth surfaces affected by decay over 20 years**

| **Disease experience** | **Number of tooth surfaces affected over 20 years (undiscounted)** | | |
| --- | --- | --- | --- |
|  | **W/O** | **With** | **Reduction from water fluoridation** |
| Decayed (untreated) surfaces | 2,963,000 | 2,116,000 | -847,000 |
| Missing surfaces* | 40,463,000 | 28,260,000 | -12,203,000 |
| Filled surfaces | 50,591,000 | 34,998,000 | -15,592,000 |
| Decayed, missing or filled surfaces^†^ | 94,017,000 | 65,375,000 | -28,642,000 |

*Each missing tooth is counted as three surfaces.

^†^The total will include some cases where one tooth surface is affected more than once: each tooth surface could be untreated, filled, then missing.

**Table E. Estimated number of teeth affected by decay over 20 years**

| **Disease experience** | **Number of teeth effected over 20 years (undiscounted)** | | |
| --- | --- | --- | --- |
|  | **W/O** | **With** | **Reduction from water fluoridation** |
| Decayed (untreated) teeth | 1,961,000 | 1,502,000 | -459,000 |
| Missing teeth | 13,488,000 | 9,420,000 | -4,068,000 |
| Filled teeth | 20,374,000 | 17,014,000 | -3,361,000 |
| Decayed, missing or filled teeth* | 35,823,000 | 27,936,000 | -7,887,000 |

*The total will include some cases were one tooth is affected more than once: each tooth could be untreated, filled, then missing.

**Table F. Quality-of-life values for different oral health states**

| **Health state** | **Definition** | **Health-related QoL score** |
| --- | --- | --- |
| **Low level of decay**  (DMFT 0–2) | People have an average of 0.7 teeth affected by decay, with 80 percent treated (mostly with fillings) and the remaining untreated. The untreated decay is relatively minor and is unlikely to be symptomatic (i.e. the person is unlikely to be aware of decay). | 1 |
| **Moderate level of decay**  (DMFT 3–11) | People have on average 7 teeth affected by decay, with an average of 1 tooth untreated. | 0.999 |
| **High level of decay**  (DMFT 12+) | People have an average of 21 teeth affected by decay, with 60% of decayed teeth having fillings, and 37% missing and the remainder untreated. The average number of teeth with untreated decay is 1 (same as for the moderate group). | 0.997 |
| **Edentulism (no teeth)*** | Assumed to have the same quality of life as people with a high level of decay. | 0.997 |

*Same proportion of people with and without fluoridation, i.e. no impact on results.
